# Low Power Consumption Silica Thermo-Optic Switch Based on Polymer Cladding

**DOI:** 10.3390/polym17233214

**Published:** 2025-12-02

**Authors:** Tianyu Zhong, Jiale Qin, Wenqian Liu, Yuqi Xie, Chahao An, Yinxiang Qin, Yunji Yi

**Affiliations:** 1 College of Integrated Circuits and Optoelectronic Chips, Shenzhen Technology University, Shenzhen 518118, China; zhongtianyu2023@email.szu.edu.cn (T.Z.); 202201202022@stumail.sztu.edu.cn (Y.Q.); 2 College of New Materials and New Energies, Shenzhen Technology University, Shenzhen 518118, China

**Keywords:** optical switch, heterogeneous hybrid integration, polymer cladding, photonic integrated chip

## Abstract

Silica-based splitters, couplers, and arrayed waveguide gratings are key components in optical communication. However, the high tuning power consumption of silica chips limits their development and application in fields such as Reconfigurable Optical Add/Drop Multiplexers and Mode Division Multiplexing. In this work, we demonstrate a silica thermo-optic switch based on polymer cladding within a Mach–Zehnder Interferometer framework, in which a UV-curable polymer is employed as the upper cladding to enhance thermal efficiency. The device exhibits a power consumption of 48 mW, rise and fall response times were 215 µs and 271 µs, compared to all-silicon switches, the power consumption is reduced by 75%, and the switching speed is improved by nearly a factor of two, while maintaining a comparable insertion loss. Experimental results demonstrate an insertion loss of 8.53 dB and an extinction ratio of 10.12 dB.

## 1. Introduction

With the explosive development in communication technology, few-mode optical fibers and mode-division multiplexing photonic chip technologies have emerged as research hotspots, capable of significantly enhancing information transmission and processing capabilities without incurring additional process costs. In the optical communication domain, silica-based devices are crucial because unlike higher-integration silicon chips, silica offers lower cost, reduced fiber mode mismatch loss, and lower transmission loss [1,2,3,4].

According to the different working principles, there are thermo-optic switches and electro-optic switches. Although electro-optic switches have fast response times and can achieve high-speed optoelectronic conversion for rapid optical networks, they currently suffer from losses due to carrier absorption, the cost of electro-optic materials, and the stability of high-performance electro-optic materials. In contrast, thermo-optic switches offer high energy conversion efficiency, low insertion loss, and low cost, making them become a vital part of LSI [3].

At present, a series of thermo-optic switches with a variety of waveguide materials such as silicon on insulators (SOI), silica, and polymers have been reported. However, in optical communication, optical fiber is still the main carrier of the optical path [4]. Silica waveguide has a lower coupling loss attributed to the smaller refractive index difference with optical fibers. Additionally, silica waveguides exhibit lower path loss and greater device stability, enabling them to better fulfill the demands of Industrial Commercialization [5].

Silica-based optical switches are key components for switching and regulating optical paths in mode-division multiplexing. In 2014, Katayose et al. employed triplebranched spot-size converters (SSCs) and heat insulating grooves to reduce coupling loss and switching power; they obtained a low insertion loss of 3.6–6.8 dB and a low power consumption of 1.6 W [6]. Afterwards, in 2023, they introduced a model-selective thermo-optic switch based on silicon dioxide; the power consumption was 433.24 mW, its rise and fall times of 560.9 µs and 794.8 µs, respectively [7]. In 2024, they proposed a multimode reconfigurable thermo-optic switch array; the 2 × 2 MZI switch unit demonstrated a power consumption of 404.82 mW [8]. To reduce the driving power consumption, researchers have structurally enhanced the utilization of the thermal field by introducing air trenches on both sides of the silica. In 2024, Manzhuo Wang et al. developed a silica-based thermo-optic switch with air trenches. The power consumption was 315.8 mW, representing a 40% reduction compared to same switches without air trenches [9]. In the latest paper, due to advancements in fabrication technology and structural optimization, the average power consumption of the thermo-optic switch has been reduced. Guoyan Zeng et al. design the switch which comprises the multimode interferometer; the rise and fall times of the switch are 0.88 and 0.94 ms, respectively, with a maximum power consumption of 246.6 mW [10].

Polymer’s low thermal conductivity confines heat near the waveguide, and its high thermo-optic coefficient (−1 × 10^−4^/K), ten times greater than that of silica, consumes less driving power than all-silica switches. In addition to hybrid core integration, Hybrid-Integrated thermo-optic switch with polymer cladding and silica waveguide was also investigated. In 2019, Ming-hui Jiang reported a polymer/silica hybrid waveguide switch, in which both the waveguide and the upper cladding are made of polymer. The switch exhibits a low switching power of 5.2 mW, with measured rise and fall times of 192.2 µs and 201.1 µs, respectively, and an insertion loss of approximately 12 dB [11]. Afterwards, in 2022, Xie Yuqi et al. discussed a thermo-optic switch combining a polymer cladding with air trenches. By introducing air trenches and adjusting the waveguide core width, the device’s power consumption was theoretically reduced from 115 mW to 5.7 mW [12]. The study also highlighted that combining polymer imprinting technology with air trenches could significantly lower device costs. Although Xie et al. [12] demonstrated a hybrid polymer–silica switch, this work relied on hypothetical polymer, and the device performance was only theoretically analyzed. In contrast, the present work is the first experimental demonstration of the silica thermo-optic switch based on polymer cladding, including full fabrication and dynamic switching measurements.

The introduction of the polymer cladding enhances the efficiency of heat utilization. Specifically, the thermal conductivity of the polymer (0.2 W/m·K) is significantly lower than that of silica, which suppresses lateral heat diffusion and concentrates the generated heat in the waveguide core region. In addition, the polymer exhibits a thermo-optic coefficient that is 1–2 orders of magnitude larger than that of silica, which directly enhances the phase-shift efficiency. These two combined effects lead to improved heat utilization and reduced switching power. In addition, compared with traditional silica-based structures, the introduction of the polymer cladding contributes to the reduced response time observed experimentally.

In this work, we use polymer as a cladding for silica waveguides, offering lower absorption and higher TO coefficient; then, we fabricated a thermo-optic switch with an acrylic polymer cladding, which matches the refractive index and optical properties of optical fibers; finally, parameter testing (optical loss, thermal response, power consumption, and switching speed) is systematically evaluated on the fabricated device.

## 2. Materials and Methods

The proposed thermo-optic switch consists of a Ge-doped silica core, lower cladding silica, UV-curable polymer upper cladding (2003T7), and aluminum heater. The refractive index of the Ge-doped silica core is 1.48@1550 nm, while the upper cladding uses the UV epoxy-based polymer 2003T7, which has a fitted refractive index of 1.46@1550 nm obtained from ellipsometry measurements. The lower cladding silica has a refractive index of 1.444@1550 nm.

**Figure 1:**
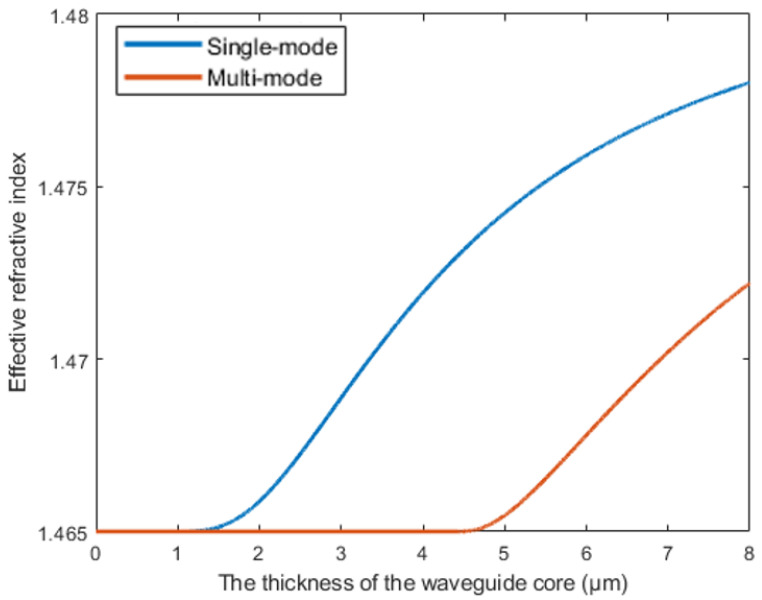
Single-mode and multi-mode modes with different widths at 1550 nm.

The contrast between the core and cladding materials ensures single-mode propagation at 1550 nm, which is the standard operating wavelength for optical communication systems. Then, Figure 1 shows the function between the thickness (the thickness of the core is square waveguide side length) of the waveguide core and mode, indicating that waveguide dimensions of 3 × 3 µm can support single-mode transmission.

**Figure 2:**
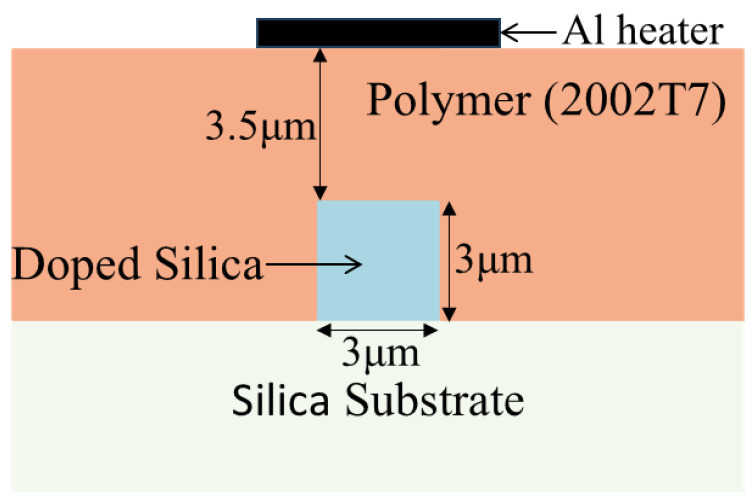
The schematic of the cross section of the switch.

**Figure 3:**
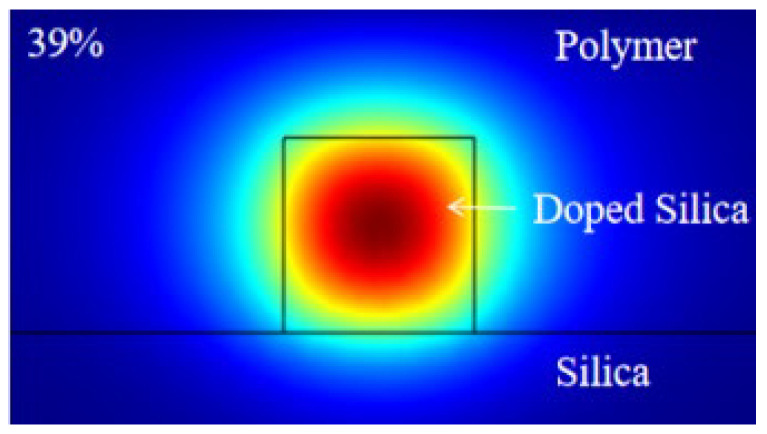
The optical field distribution of the device.

Figure 2 shows the schematic of the cross section of the thermal-optical switch. Analysis reveals that when the spacing between the heater and the waveguide is less than 2.5 µm, metal absorption of light in the waveguide significantly increases. Small spacing results in increased light absorption by the metal, thereby raising the loss [13]. Considering the absorption by the metal electrode and the viscosity and actual thickness of the polymer cladding, we increase the spacing to 3.5 µm. The optical field distribution simulated using finite element method (FEM) is shown in Figure 3, confirming that the optical mode is well confined within the doped silica waveguide region.

**Figure 4:**
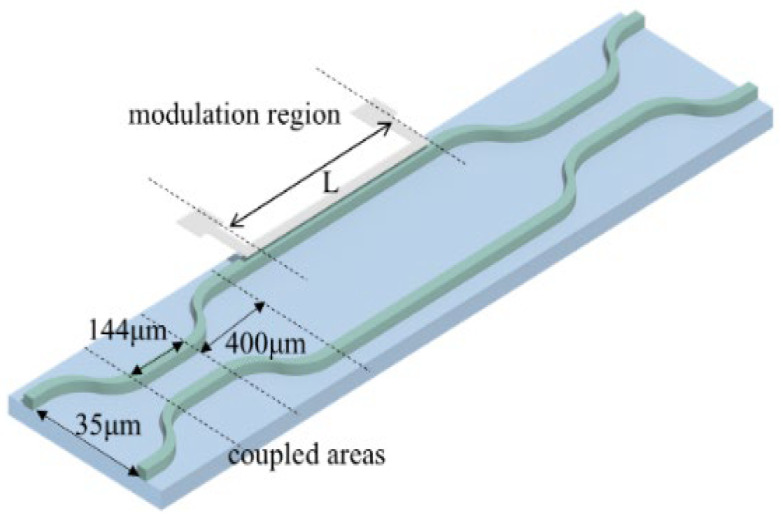
The schematic of the proposed switch structure.

The schematic of this thermal-optic switch model is shown in Figure 4. It was developed based on the MZI waveguide, consisting of two 3 dB directional coupled areas and a modulation region. Its structural parameters were designed by using the Beam Propagation Method (BPM) to make sure the light will emit to the modulation zone after passing through the coupling zone with the same power. The modulation arm length L is 7000 µm, which is designed to balance optical path losses and heating efficiency.

An aluminum electrode with a fixed thickness of 100 nm is positioned directly above the upper polymer cladding along the modulation arm. This metal layer serves as a micro-heater, locally modulating the refractive index through the thermo-optic effect. The heater length is equal to the modulation arm to maximize the overlap between the thermal field and the optical mode. FEM simulations employed steady-state heat transfer and wave optics modules to analyze both thermal distribution and optical field variation.

**Figure 5:**
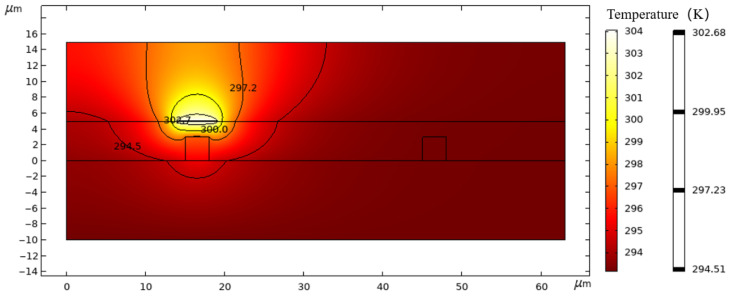
The thermal field distribution map for the modulation region.

**Figure 6:**
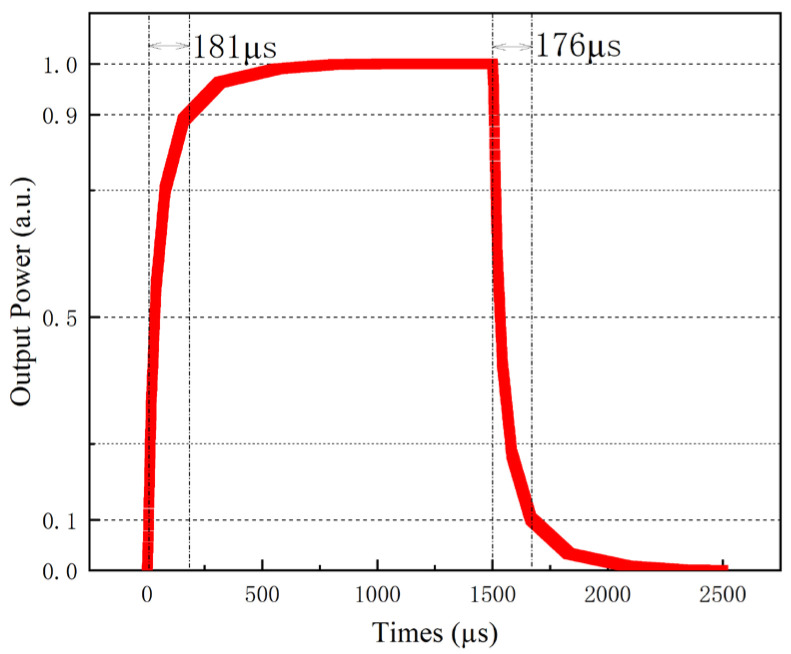
The response times for the thermal-optical switch.

**Figure 7:**
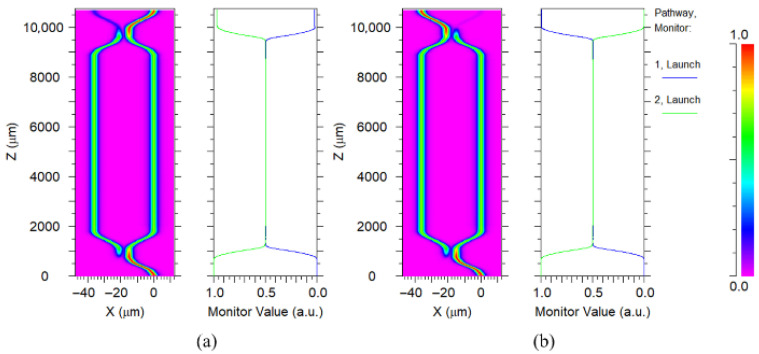
Operational state diagrams of the switch. (**a**) Bar state: without heating, the two arms remain phase-matched, and power exits the port. (**b**) Cross state: heater induced pi-phase shift causes power to be canceled.

The thermal field distribution map, as delineated in Figure 5, offers a detailed visualization of the temperature gradients across the thermal-optical switch, which is instrumental in analyzing the parameters of this device. Dynamic thermal simulations show that the device can reach its steady-state temperature within a few hundred microseconds. Through the structure of the switch and its specific parameters, we conducted FEM using the simulation package to calculate its performance metrics; the switch operates with a power consumption of 37.9 mW, the rise time and fall time of the switch are distributed as 181 µs and 176 µs, as shown in Figure 6. Finally, the switching operation of the device was simulated using the beam propagation method (BPM). When no heating current is applied to the electrode, the optical power emerges from the right port, corresponding to the bar state. Conversely, when the electrode is heated, a thermo-optic phase shift is induced and the optical power is redirected to the opposite port, corresponding to the cross state, demonstrating clear light transfer between bar and cross ports under thermal modulation, illustrating the bar state Figure 7a and cross state Figure 7b of the switch.

## 3. Results and Discussion

**Figure 8:**
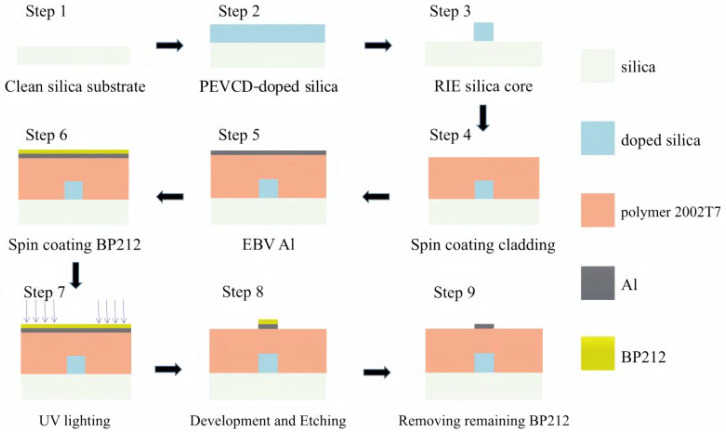
The schematic diagram of the fabrication process of the thermal-optic switch.

**Figure 9:**
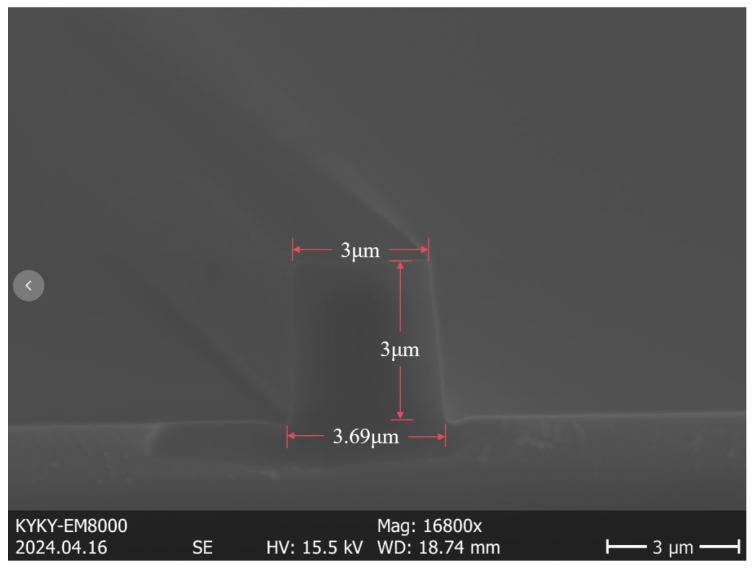
The SEM image of the etched doped silica core showing trapezoidal geometry.

The specific manufacturing process of the thermal-optic switch is depicted in Figure 8. We initially deposited a 10 µm-thick layer of silicon dioxide on a 6-inch silicon substrate by Plasma Enhanced Chemical Vapor Deposition (PECVD). The Ge doping process is employed to achieve the desired refractive index of 1.48 and a thickness of 3 µm for the core layer. After deposition, standard photolithography and Reactive Ion Etching (RIE) were used to define the waveguide pattern. The mask pattern contained the MZI configuration with two 3 dB directional couplers and two interferometric arms. The 3D core layer of the thermo-optic switch, under the SEM scan, as shown in Figure 9, displays a trapezoidal geometry with a top width of 3.0 µm, a bottom width of 3.69 µm, and a height of 3.0 µm. This geometric deviation from the designed square section originates from the etching anisotropy of the silica process, leading to slightly tilted sidewalls.

Subsequently, the polymer cladding spun coated at 6000 rpm to form a 6.5 µm-thick cladding layer after UV curing. To avoid oxygen inhibition during curing, the polymer layer was UV-cured under a nitrogen atmosphere using an exposure intensity of 500 mW/cm^2^ for 25 s at room temperature. This nitrogen-assisted curing was essential because the 2003T7 polymer is oxygen-sensitive; in the presence of oxygen, high-dose or deep UV irradiation can lead to photo-oxidation at the surface, forming a hard “oxygen-inhibited crust.” Such crusts have different shrinkage rates compared to the underlying polymer, which can induce microcracks during post-curing or thermal cycling. The nitrogen protection effectively eliminates this issue and ensures high optical uniformity and mechanical stability of the cured polymer layer.

**Figure 10:**
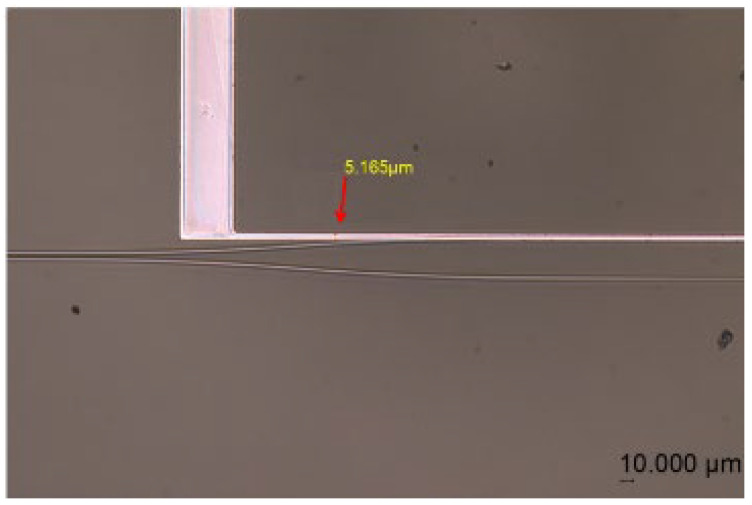
The electrodes fabricated in the switch modulation area under microscope, with a measured width of approximately 5 µm.

For metal heater fabrication, an aluminum thin film, with a thickness of 100 nm, is deposited on the cladding surface using an electron-beam evaporation system (EBS-99) with a standard Al evaporation process for the next step involving the fabrication of heating electrodes. Followed by spin coating BP212 positive photoresist, the photolithography of electrode patterns was performed using the SUSS MA Gen4 (SUSS MicroTec SE, Garching, Germany), operating at a wavelength of 365 nm (UV), with an exposure setting of 200 mJ/cm^2^ and a duration of 4 s after pre-baking at 90 °C for 10 min. Then, a 0.5% NaOH solution was chosen as a developing liquid that removes exposed BP212 while etching for unwanted aluminum metal film. Ultimately, the electrode patterns were successfully obtained as depicted in Figure 10. The excellent adhesion between the 2003T7 polymer and the aluminum film ensured uniform heating and reliable electrical contact, crucial for achieving consistent switching behavior.

**Figure 11:**
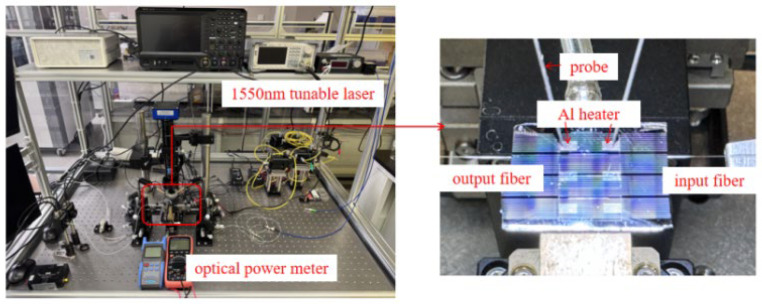
The switch test platform and device test magnification diagram.

This fabricated switch is tested using the test platform on the optical vibration isolation platform, as shown in Figure 11. Initially, with the probe in contact at both electrode terminals, a heating resistance of 1.6 kΩ was measured. Characterization of the thermal-optic switch was conducted by launching a 1550 nm wavelength light signal from a tunable laser into the input waveguide and measuring the output power with an optical power meter. Dynamic characterization was performed by applying voltage to the heating electrodes and gradually increasing the driving voltage. A sinusoidal power transformation was observed at output ports.

(1) $$P=\frac{V^{2}}{R}$$

**Figure 12:**
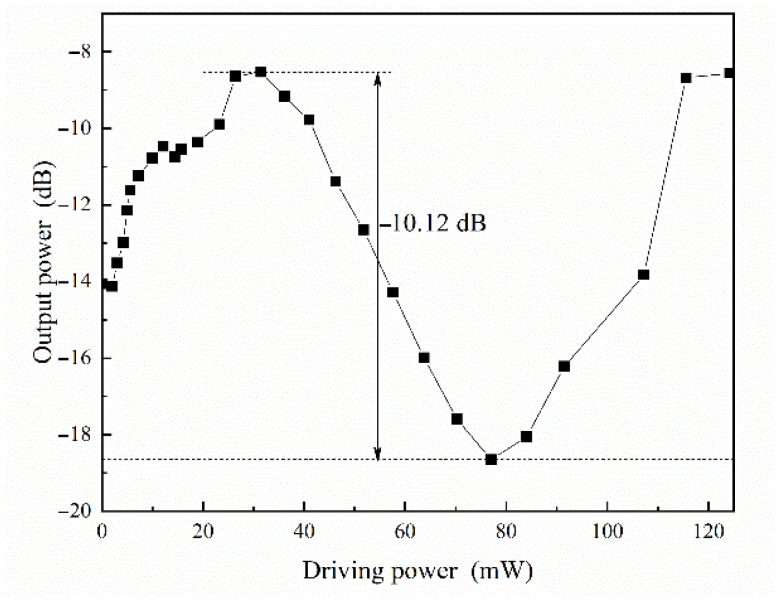
The light intensity transmission of the switch by increasing the modulation power.

After converting the voltage to power using Equation (1), the power curve is shown in Figure 12. As observed in Figure 12, the switch exhibited the insertion loss of 8.53 dB. In the device fabrication process, we used direct facet cleavage. Considering that each facet typically contributes 2 dB, the actual transmission loss of the device is only 4.53 dB, which is consistent with previously reported losses for silica waveguide devices. This loss mainly comes from scattering at the interfaces of the silica core, polymer cladding, and fabrication, with the extinction ratio of 10.12 dB. With a measured heating metal resistance, the switch’s driving power consumption was calculated to be 48 mW.

**Figure 13:**
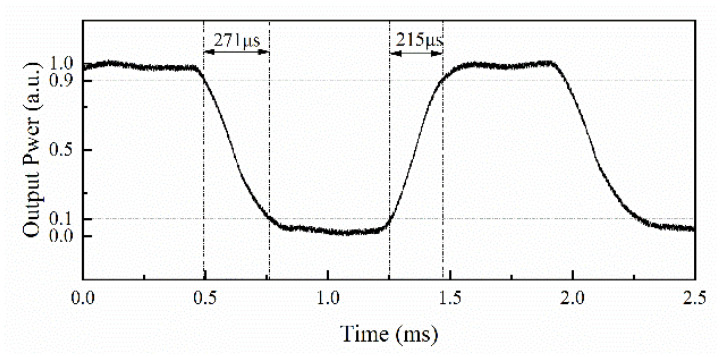
The response time of the proposed switch.

Further measurements were conducted to determine the switch’s response time by using a signal generator as a driver and monitoring the output with an oscilloscope. Figure 13 shows the oscilloscope image of the input driving signal and output light signal measurements. By employing an 8 V square wave driving signal with a duty cycle of 0.5, the measured rise and fall times at output port 1 were 215 µs (10–90%) and 271 µs (90–10%), respectively.

Furthermore, it can be observed that in the initial state, the port did not emit at its maximum power capacity, mainly affected by manufacturing process and refractive index difference. From Figure 9, the actual waveguide cross section is a trapezoid with a top width of 3 µm, a bottom width of 3.69 µm, and a height of 3 µm. This does not match the simulated device cross section.

Adjusting the initial phase is crucial, primarily due to the following two factors: waveguide etching width and cladding refractive index. Etching can narrow the waveguide width by ~0.5 µm. Although we designed compensation to minimize this error, actual widths are still inaccurate because we cannot precisely determine the specific values in large quantities. Our BPM analysis confirmed that different widths (within a 0.5 µm range) cause phase shifts, which align with experimental results. While this analysis was not included in the text due to space limits, adjusting width compensation parameters can improve phase point adjustment in the future. On the other hand, the cladding refractive index, determined by ellipsometry, also has potential errors. Our simulations using ellipsometry data replicated the experimental results. Based on waveguide width observations via electron microscopy, we believe factor waveguide width is the main contributor. We have incorporated these actual dimensions into our simulations and the BPM software analysis confirmed that the actual manufacturing process causes huge phase shifts, which align with experimental results.

The results of the thermo-optic switch in this study were compared with those of other thermo-optic switches based on different material as reported in the literature, as shown in Table 1. The table lists the core structural configurations, operation wavelengths, insertion losses (IL), driving power consumption (PC), and response times (RT) of various thermo-optic switches. It can be observed that the use of the polymer as the upper cladding, complementing the silica waveguide, can reduce the power consumption of such silicon-based thermo-optic switches.

To enable a quantitative comparison with prior art, we compared our switch against previously reported devices that employ a comparable all-silica material system and Mach–Zehnder interferometer (MZI) architecture. As summarized in Table 1, the selected reference devices correspond to works [7,8,10,14]. These switches exhibit an average power consumption of 320 mW. Although certain related switches in the broader literature achieve lower power consumption, we have adopted a conservative estimate of 75% reduction in the revised manuscript. This percentage is explicitly derived from the average value of the four directly comparable devices listed above and the switching time has been reduced by half.

Overall, the quantitative comparison in Table 1 clearly demonstrates that the integration of a polymer upper cladding provides a highly effective means of improving both static and dynamic performance metrics. The hybrid configuration not only minimizes thermal losses but also maintains reasonable optical performance, validating its suitability for next-generation low-power photonic integrated circuits (PICs). The balance between optical efficiency and thermal control achieved in this work represents a significant step toward practical deployment of energy-efficient reconfigurable optical interconnects.

## 4. Conclusions

In summary, we have designed and fabricated a silica MZI thermo-optic switch incorporating a polymer cladding. The device exhibits an insertion loss of 8.53 dB, an extinction ratio of 10.12 dB, and a driving power consumption of 48 mW for state switching. Dynamic testing reveals rise and fall times of 215 µs and 271 µs, respectively. This switch leverages a silica platform, facilitating seamless integration with other silica-based devices. Compared to all-silica switches, our hybrid design achieves a 75% reduction in power consumption and nearly doubles the switching speed, while maintaining comparable insertion loss. These enhancements stem from the synergistic integration of polymer and silica materials, which improve thermal efficiency through the polymer’s low thermal conductivity and high thermo-optic coefficient.

Future efforts will focus on investigating advanced polymer formulations for additional power savings and performance optimization. We also plan to incorporate air trenches to minimize heat dissipation, further lowering power consumption and enhancing thermo-optic tuning efficiency. Overall, this work advances the development of energy-efficient reconfigurable optical components for applications in optical communication systems.

## Figures and Tables

**Table 1:** Thermal optical switch critical parameter of a different structure.

References	Structure (Upper Cladding/Core/Under Cladding)	Wavelength (nm)	IL (dB)	PC (mW)	RT (µs)
[6]	silicon/doped silica/silica	1550	5	1600	1200
[7]	silica/doped silica/silica	1550	4.56	433.24	650
[8]	silica/doped silica/silica	1550	~3	300	509.7
[10]	silica/doped silica/silica	1550	2	246.6	900
[14]	silica/si/silica	1550	/	300	2000
[11]	PMMA/SU-8/silica	1550	13	5.2	197
[12]	Polymer/Doped Silica/Silica	1550	/	~5.7	224
This work	Polymer(2002T7)/doped silica/silica	1550	8.53	48	250

## Data Availability

The original contributions presented in this study are included in the article. Further inquiries can be directed to the corresponding author.
